# Supplementary material for: Trends in snakebite deaths in India from 2000 to 2019 in a nationally representative mortality study
Source: eLife. 2020 Jul 7;9:e54076. doi: 10.7554/eLife.54076 (PMC7340498; doi:10.7554/eLife.54076)
Supplement: Transparent reporting form [file elife-54076-transrepform.docx]

***eLife’s* transparent reporting form**

We encourage authors to provide detailed information *within their submission* to facilitate the interpretation and replication of experiments. Authors can upload supporting documentation to indicate the use of appropriate reporting guidelines for health-related research (see [EQUATOR Network](http://www.equator-network.org/%20)), life science research (see the [BioSharing Information Resource](https://biosharing.org/)), or the [ARRIVE guidelines](http://www.plosbiology.org/article/info:doi/10.1371/journal.pbio.1000412) for reporting work involving animal research. Where applicable, authors should refer to any relevant reporting standards documents in this form.

If you have any questions, please consult our Journal Policies and/or contact us: [editorial@elifesciences.org](mailto:editorial@elifesciences.org).

**Sample-size estimation**

- You should state whether an appropriate sample size was computed when the study was being designed
- You should state the statistical method of sample size computation and any required assumptions
- If no explicit power analysis was used, you should describe how you decided what sample (replicate) size (number) to use

Please outline where this information can be found within the submission (e.g., sections or figure legends), or explain why this information doesn’t apply to your submission:

Sample size estimation was not conducted for this study as it was not applicable to our study methods, which utilized nationally representative data from India’s Sample Registration System between 2001 and 2014. Further details can be found in the ‘Materials and Methods’ section and MDS study design published elsewhere.

**Replicates**

- You should report how often each experiment was performed
- You should include a definition of biological versus technical replication
- The data obtained should be provided and sufficient information should be provided to indicate the number of independent biological and/or technical replicates
- If you encountered any outliers, you should describe how these were handled
- Criteria for exclusion/inclusion of data should be clearly stated
- High-throughput sequence data should be uploaded before submission, with a private link for reviewers provided (these are available from both GEO and ArrayExpress)

This study does not involve in replication of the experiment. Snakebite mortality cases ascertained in this study were identified through dual physician assignment of cause of death in a verbal autopsy survey. Snakebite incidents are from carefully designed systematic review of published studies. Inclusion and exclusion criteria for studies included in our systematic review are described in the supplementary appendix. Additional details regarding inclusion and exclusion of snakebite deaths are described in the ‘Methods’ section.

Please outline where this information can be found within the submission (e.g., sections or figure legends), or explain why this information doesn’t apply to your submission:

**Statistical reporting**

- Statistical analysis methods should be described and justified
- Raw data should be presented in figures whenever informative to do so (typically when N per group is less than 10)
- For each experiment, you should identify the statistical tests used, exact values of N, definitions of center, methods of multiple test correction, and dispersion and precision measures (e.g., mean, median, SD, SEM, confidence intervals; and, for the major substantive results, a measure of effect size (e.g., Pearson's r, Cohen's d)
- Report exact p-values wherever possible alongside the summary statistics and 95% confidence intervals. These should be reported for all key questions and not only when the p-value is less than 0.05.

Please outline where this information can be found within the submission (e.g., sections or figure legends), or explain why this information doesn’t apply to your submission:

Statistical methods used in this study are described in the ‘Methods’ section and in the supplementary appendix. Further detail regarding specific analyses are provided in figure and table captions, where necessary. The inherent variation of our estimates are not the underline demographic estimates but determination of the causes of death. Therefore we use a plausible ranges based on independent cause assignment at central medical evaluation of causes deaths as explained in Table 1 caption 4.

(For large datasets, or papers with a very large number of statistical tests, you may upload a single table file with tests, Ns, etc., with reference to sections in the manuscript.)

**Group allocation**

- Indicate how samples were allocated into experimental groups (in the case of clinical studies, please specify allocation to treatment method); if randomization was used, please also state if restricted randomization was applied
- Indicate if masking was used during group allocation, data collection and/or data analysis

Please outline where this information can be found within the submission (e.g., sections or figure legends), or explain why this information doesn’t apply to your submission:

Since this study is an observational study, formal group allocation or masking does not apply.

**Additional data files (“source data”)**

- We encourage you to upload relevant additional data files, such as numerical data that are represented as a graph in a figure, or as a summary table
- Where provided, these should be in the most useful format, and they can be uploaded as “Source data” files linked to a main figure or table
- Include model definition files including the full list of parameters used
- Include code used for data analysis (e.g., R, MatLab)
- Avoid stating that data files are “available upon request”

Please indicate the figures or tables for which source data files have been provided:

Tables and figures are embedded within the article file submitted through the eLife portal. Source data files have been provided for figures; the data availability link for Figure 1 described in the figure caption. Datasets for Figure A1 and Figure 2 (a:b) in Excel format are uploaded into the eLife portal. Figure 3 uses the data listed in Appendix Table B1.
